# Behavior change interventions for dangerous driving behavior in low- and middle-income countries: a systematic review of interventions and outcome measurement instruments

**DOI:** 10.3389/fpubh.2025.1597331

**Published:** 2025-06-03

**Authors:** Saen Fanai, Eunice Okyere, Kissinger Marfoh

**Affiliations:** ^1^College of Medicine, Nursing and Health Science, Fiji National University, Suva, Fiji; ^2^School of Public Health, College of Medicine, Nursing and Health Science, Fiji National University, Suva, Fiji

**Keywords:** behavior change interventions, dangerous driving behavior, risky driving behavior, road safety, outcome measurement, randomized control trial

## Abstract

**Objective:**

This systematic review aims to assess Behavior Change Interventions (BCIs) targeting dangerous driving behaviors (DDBs) in low- and middle-income countries (LMICs), evaluate their effectiveness, and explore the outcome measurement instruments employed in these contexts.

**Method:**

A comprehensive search was performed across key databases such as Scopus, PubMed, CINAHL, Medline, ProQuest, Cochrane Library, and Research4life, focusing on studies published post-2015 on BCIs for DDB using randomized controlled trials (RCTs), quasi-experiments and mixed method designs. Data extraction centres on the types of intervention, theoretical frameworks, outcome measurement, and intervention effectiveness. The findings were analyzed through a narrative synthesis approach.

**Results:**

Fifteen studies were analyzed in this review, which examined BCIs aimed at enhancing driver behavior. Some interventions included public awareness campaigns, peer mentorship programs, driving courses for skill enhancement, and in-vehicle monitoring systems for safety measures. The study results revealed that widely employed behavior change theories such as the Theory of Planned Behavior (TPB), along with Social Cognitive Theory (SCT) and the Health Belief Model (HBM), were prevalent in these interventions. Surveys like the Driver Behavior Questionnaire (DBQ) and assessments of road safety knowledge were frequently used to measure behavioral changes among participants. Objective criteria included the use of GPS tracking devices, as well as the examination of insurance claims and traffic violation reports to evaluate the interventions’ effectiveness. The study focused on methods like peer influence implementation and fear-based communication strategies paired with personalized feedback, which were identified as successful approaches in the review report. However, it emphasized a lack of uniformity in utilizing validated tools for outcome measurement across various socioeconomic contexts, particularly in LMICs.

**Conclusion:**

Behavioral interventions informed by theories have demonstrated effectiveness in improving driving behaviors; nonetheless, recognized and validated measures for assessing results in lower—and middle-income settings remain unclear. In the future, research efforts should prioritize developing assessment tools that resonate with cultures and conducting studies to evaluate enduring changes in behavior. This systematic review may guide policymakers, and public health experts engaged in developing road safety programs.

**Systematic review registration:**

www.crd.york.ac.uk/prospero, identifier 578081.

## 1 Introduction

Since the mid-20th century, reports have indicated some decline in road traffic deaths and injuries due to public health initiatives, preventive efforts, and technological improvements, including safer vehicles and roads, as well as improved emergency services and trauma care (1, 2). Despite these improvements, the issue remains important, particularly within health and social care and its escalating costs for health and welfare systems across the globe and in particular low and middle-income countries (LMICs) (3). Vehicle crashes and related injuries and deaths are not random incidents; Human behavior is the critical factor in more than 90% of crashes (1). It is essential to use a multi-disciplinary framework to understand the underlying psychological influences on driving behavior and the development and evaluation of interventions to achieve improvements in road user behavior (4). In recent years, there has been a growing focus on finding effective behavior change strategies to improve various forms of driver behavior across a wide range of road user-profiles and cultural contexts (5). Empirical findings show that successful behavior change strategies involve making drivers aware of the errors that lead to accidents using persuasive communications and penalty and reward systems while supporting them in adopting preventive strategies that maintain a certain driving quality during exposure (6). Nonetheless, the field of driving behavioral change holds several challenges. First, the definition of safe driving itself is far from clear and straightforward (4). Second, the readiness of individuals to adhere to driving quality can vary greatly depending on the context (7). Notable progress has been made in advancing empirical frameworks that effectively support the formulation of targeted actions. Information gathered from actual driving behaviors has enabled researchers to assemble detailed evidence regarding the impact of individual differences on changes in driver behavior (5).

### 1.1 Background and rationale

Traveling on the roads in our society poses a considerable health danger. Every year, traffic accidents result in around 1.24 million deaths worldwide, and between 20 to 50 million individuals sustain injuries or disabilities (2). The public health burden of traffic-related injuries is particularly pronounced in LMICs, where infrastructure, enforcement, and health system responses are often limited. This review focuses on these settings to assess context-specific challenges and intervention effectiveness. Despite a large body of research identifying a great deal of DDBs, which increases the risk of traffic crashes and other adverse outcomes, a relative lack of consensus about how to change such behavior effectively still exists (8). The complex nature of DDB may contribute to the diversity of its associated interventions, including policy and enforcement interventions, BCIs, and modifications of the driving environment (1). Earlier reviews on changing driver behavior mainly approach the field from a general road safety perspective, do not systematically focus on DDB, and do not specifically describe the extent to which studies included in those reviews employed intervention content derived from psychological theories, models, and insights (6). While previous research indicates that varying levels of cognitive constructs help to predict dangerous driving, these predictions should only serve as starting points for theory-based BCIs, as psychological theory tends to be abstract (5).

Previous research has highlighted that studies focusing on DDB often fail to incorporate relevant theories or models when developing interventions aimed at changing this behavior. For instance, one review of alcohol-related driving offenders was based on the characteristics of actual offenders rather than their risky driving behaviors (9), and another review classified various dangerous behavior characteristics based on *a priori* risk implications and then categorized some of these behavior characteristics into intervention types, either informational, technical, training, legal regulation, persuasion, rewards, sanctions, or combinations (10). Trying to change DDB in this manner has thus far met with mixed success. It might only contribute to mild effects because the BCIs are hypothesized based on identified risky driving-related behaviors and consequences. Still, no explicit theoretical framework was used to explain the nature of the predicted associations between the intervention content, behavioral determinants, and behavior (5). Commonly, individual-level BCIs neglect the broader contextual and cognitive factors influencing long-term change (7).

Previous reviews found that the most studied behavior change techniques included feedback and monitoring, goal setting and planning, social support, knowledge sharing, and natural consequences. All interventions proved somewhat effective in reducing DDB (5, 7). Goal setting, penalties, and vicarious experience were most frequently reported to create an overall change in driving Behavior (11). The most frequently reported outcome measures used to assess the effectiveness of the interventions were accidents and injuries, self-reported confidence, self-reported driving violations, behavior or sanctions, self-reported ability concerning driving, and self-reported behavioral change (4, 5).

### 1.2 Objective of the review

This review’s primary aim is to evaluate BCIs to reduce DDB in LMICs and synthesize context-specific evidence on their effectiveness and evaluation methods.

The findings of this review can assist policymakers and practitioners in creating innovative BCI tools that effectively address the needs of modern drivers (6).

### 1.3 Scope and definition of dangerous driving behavior

DDB consists of risky actions or patterns that significantly increase the likelihood of road accidents, resulting in injuries and fatalities. The definitions from the World Health Organization, Elander et al., and Reason et al. (2, 12, 13) inform our understanding of DDB, which includes at least the following behaviors:

- Speeding: Operating a vehicle above the legal speed limit or too fast for road or traffic conditions.
- Distracted driving: Activities that divert attention from driving, such as mobile phone use and interactions with passengers.
- Driving under the influence of psychoactive substances: Operating a vehicle while impaired by alcohol, drugs, or other substances.
- Fatigued driving: Driving while drowsy, often due to lack of sleep or prolonged hours on the road.

The established definition serves as our inclusion criteria for identifying relevant outcomes, intervention targets, and measurement instruments in the studies. Our analysis employs this definition to synthesize intervention effectiveness and the appropriateness of outcome measures across various contexts.

### 1.4 Significance of studying behavior change intervention

When appropriate BCI models and tools for measuring these interventions are developed, there can be a significant improvement in traffic safety, a reduction in the severity of DDB, and the prevention of traffic accidents before they occur (1, 4). BCIs that focus on reducing dangerous driving are crucial as they seek to modify social behaviors. These interventions promote social learning and experiential learning through various real-world tasks and simulations provided to participants. As alluded to, the findings of this review can assist policymakers and practitioners in creating innovative BCI tools that effectively address the needs of modern drivers (6).

## 2 Method

### 2.1 Information sources and search strategy

This systematic review was conducted and documented in compliance with the Preferred Reporting Items for Systematic Reviews and Meta-Analyses (PRISMA) guidelines (14).

A comprehensive search strategy was developed using a combination of keywords and Medical Subject Headings (MeSH) terms related to dangerous driving BCIs, and outcome measurement instruments. The strategy was adapted for each database. The research supervisors, who are seasoned researchers who have led researchers on various health-related research projects, reviewed the strategy (15). The final search was conducted on September 14, 2024, and included seven electronic databases: Scopus, PubMed, CINAHL, Medline, ProQuest, Cochrane Library, and Research4life. Every database underwent an individual search, and the search approach for Medline, one of the databases, can be found in Table 1. The search strategy utilized a combination of controlled keywords and subject headings. An example of that used in Research4life and PubMed include: (“dangerous driving Behavior” OR “risky driving Behavior” OR “unsafe driving” OR “reckless driving”) AND (“Behavior change intervention” OR “Behavioral intervention” OR “educational program” OR “training program” OR “policy intervention”) AND (“outcome measurement” OR “evaluation” OR “assessment” OR “instrument”). The search was restricted to English-language publications involving human subjects and published from 2015. The search terms were aligned with MeSH headings whenever feasible. The reference lists of included studies and pertinent systematic reviews were also reviewed to identify additional eligible studies.

**Table 1:** Behavior change intervention study characteristics.

Authors	Geographic region	Study year	Type of BCI	Study design	Target population	BC theory	Outcome measures
Abdul- Wahab et al. (32).	Nigeria	2016	Road safety education and training	MM-descriptive survey and FGD	Commercial drivers	Health Belief Model (HBM)	Knowledge, compliance and attitudes
Cutello et al. (24).	United Kingdom	2021	Comparison of fear-based and positively framed road safety films	Randomized control trial	General vehicle drivers	Fear appeal theory and social cognitive theory	Emotional arousal and risky driving behavior
Fowode et al. (25).	Nigeria	2023	Road safety education and first aid training intervention	Quasi experiment	University drivers	Social Cognitive Theory (SCT)	Road safety knowledge
Habyarimana et al. (27).	Kenya	2015	Sticker messaging intervention and Radio campaign	Randomized control trial	Mini-bus drivers	Social Norm theory and Collective Action Theory	Accidents and insurance claims
Jaensirisak et al. (34).	Thailand	2020	Road safety Education campaigns and workshops	Quasi experiment	Motorcyclist	Theory of Planned Behavior (TPB)	Risky driving behaviors
Mabayoje et al. (35).	Nigeria	2022	Education enlightenment campaign	Quasi experiment	Commercial minibus drivers	Social Cognitive Theory (SCT)	Understanding of traffic rules and risky driving behavior
Mohamed et al. (36).	Egypt	2018	Traffic safety awareness program	Quasi experiment	General drivers	Health Belief Model (HBM)	Knowledge of traffic safety practices
Nadimi et al. (23).	Iran	2021	Classroom theory education and in-car practical training	Structural Equation Modelling (SEM)	General drivers	Social Cognitive Theory (SCT)	Dangerous driving behavior, including crash frequency and traffic violations
Nthoki et al. (26).	Kenya	2024	Information, Education and Communication (IEC) initiative	Mixed method convergent parallel design	Motorcyclists	Social Cognitive Theory (SCT)	Road safety knowledge and behavior change
Nwadinigwe et al. (37).	Nigeria	2018	Road safety education program	Descriptive survey	Commercial vehicle drivers	Health Belief Model (HBM)	Knowledge and risky driving behavior
Mazengia et al. (33).	Ethiopia	2023	In-depth interviews using an open-ended interview guide to understand perceptions of risky driving behaviors	Qualitative study using thematic analysis	10 public transport drivers, 4 driving school instructors, 3 traffic police officers	Health Belief and Social Cognitive Theory (HBM/SCT)	Understanding perceptions of risky driving behaviors
Okafor et al. (31).	Nigeria	2015	Health and safety education	Quasi experiment	Commercial mini-bus drivers	Theory of Planned Behavior (TPB)	Knowledge and adherence to road safety rules
Campbell et al. (28).	Tanzania	2022	SMS text messaging reminders.	Randomized control trial	Commercial motor cycle taxi drivers	Theory of Planned Behavior (TPB)	Adherence to helmet use
Onuka et al. (29).	Nigeria	2015	Public education campaign	Ex-post facto design	Commercial vehicle drivers	Theory of Planned Behavior (TPB)	Compliance with traffic rules and risky driving behavior
Stephan et al. (30).	Australia	2024	3 to 6-week multi-stage driving Behavior change program (P drivers program)	Randomized control trial	Novis drivers with a probationary license	Self-regulation theory and social learning theory	Risky driving behavior and traffic infringements

### 2.2 Study selection

Per the recommended approach for systematic reviews, three independent reviewers (Julianne Borugu, Clelia Raubebe and Susan Tavimele) assessed the eligibility of studies for inclusion in the review. The three reviewers (JB, CR, ST) were public health researchers with training in systematic review methodology. They used a standardized screening form to apply inclusion and exclusion criteria. Reliability was ensured by independent screening and resolution of disagreements through consensus discussion, enhancing consistency in study selection. Any disparities in their findings were addressed through discussion until a consensus was reached. Initially, the search results were evaluated based on the title and abstract. The full text was obtained if the eligibility was unclear or the abstract was not available. The eligibility criteria were then used to assess the inclusion of the full-text studies in the review (16, 17).

### 2.3 Eligibility criteria

#### 2.3.1 Participants

Vehicle drivers of any age group and gender were included, regardless of health status (participants in good health or individuals with particular health issues or medical conditions).

#### 2.3.2 Interventions

Studies that involved any BCIs aimed at reducing DDB, such as educational programs, technological aids, and media campaigns, were included. Any interventions that aim to raise awareness and improve knowledge about road safety among vehicle drivers, addressing traffic rules, the dangers of risky behaviors, and proper driving techniques are included. Interventions delivered through formal classroom settings, online modules, workshops, or seminars are included. The target audiences often include new drivers, commercial drivers, or offenders enrolled in remedial driving courses. The interventions must address at least one of the following DDB: speeding, aggressive driving, distracted driving, and impaired driving, which are the leading causes of traffic accidents globally.

#### 2.3.3 Control or comparator

Studies with or without comparators (e.g., no intervention or alternative interventions) are acceptable.

#### 2.3.4 Outcomes

The behavioral change intervention had to target one of the following individual modifiable health behaviors identified by the World Health Organization as leading risk factors for road traffic accidents and injuries (2): Speeding, aggressive driving, impaired driving, distracted driving, and driving when fatigued. For inclusion in the review, the study had to report data regarding the effectiveness of Behavior change. Additionally, studies were included if they reported variables closely related to Behavior change; this included potential mediators of Behavior change (e.g., health status or physical activity self-efficacy). Studies presenting secondary outcomes data, including Road traffic accidents, injuries, adherence to traffic rules, and other related behavioral and safety outcomes, are accepted.

#### 2.3.5 Study design

The systematic review included RCTs, non-RCTs, Quasi-experimental studies, Cohort studies, Case–control studies, Cross-sectional studies, and including Qualitative studies that seek to explore insights into intervention mechanisms and contexts. Ecological studies and studies with small sample sizes (such as case studies) were considered for inclusion. Systematic reviews and Conference abstracts were not considered.

#### 2.3.6 Data collection process and data items

Data was extracted using a standardized form created specifically for this review. Data extraction for each included study was done independently by the reviewers (JB, CR, ST), and any disagreements were resolved by checking and discussing the original study until a consensus was reached. The reviewers reached a 90% consensus on data extraction, with the primary discrepancies related to studies lacking clear outcomes. Information that was extracted included Study characteristics (e.g., authors, year, country, study design), Participant characteristics (e.g., age, gender, driving experience), Intervention details (e.g., type, duration, delivery method), Outcome measures (e.g., instruments used, primary and secondary outcomes), and Results (e.g., effectiveness of interventions, measured outcomes).

#### 2.3.7 Risk of methodological Bias

The methodological quality of the included articles was assessed using the Cochrane Risk of Bias tool for RCTs (18), the ROBINS-I tool for non-randomized studies (19) and the Quality Assessment Tool for Quantitative Studies for observational studies (20). For randomized trials, the Cochrane Risk of Bias (RoB 2) tool was used; for non-randomized studies, we applied ROBINS-I; and for observational studies, we used the Quality Assessment Tool for Quantitative Studies. Two reviewers independently rated each study. Discrepancies were resolved through discussion with a third reviewer. We compiled the results from the subjective judgment matrix, which considered the authors’ conclusions, qualitative and quantitative data demonstrating statistically significant differences among participants and the BCI outcomes, and the methodological quality of the included articles. The protocol for this review overview was created before conducting the review. It was submitted to the PROSPERO International Prospective Register of Systematic Reviews. The reporting of this systematic review adhered to the Preferred Reporting Items for Systematic Reviews and Meta-Analyses (PRISMA) guidelines.

#### 2.3.8 Summary measures and synthesis of results

##### 2.3.8.1 Summary measures

To assess how well BCI can help decrease DDBs, we will use summary measures to give a structured evaluation of the results achieved in this study. The changes in Behavior will be examined by assessing the tools such as self-reported surveys and observation of the DBQ, which looks at driving actions like speeding, traffic violations and close calls. We will also look at pre and post-evaluations to gauge any changes in drivers’ understanding of traffic regulations and their adherence to them. Depending on the studies, metrics, like GPS tracking for speed compliance and records of traffic violations, will be used to observe changes in behavior compared to self-reported information. Also, tools such as the Emotional Arousal Scale will be utilized to evaluate attitudinal shifts measuring responses to safety messages and interventions. These summarized measures will be gathered for comparison across studies to help assess the effectiveness of different BCIs intended to modify driver behavior and adherence.

##### 2.3.8.2 Synthesis of results

To effectively compile and make sense of the results from the review, a narrative synthesis method involves looking at study designs and interventions while also considering the range of outcome measures used in each study. The initial categorization of BCI will be based on type, such as public awareness campaigns or driver improvement programs. This allows for comparisons between intervention approaches, like behavioral messaging and peer-supported mentoring initiatives. We will first examine the framework of each study with an emphasis on different health behavior change theories like TPB, HBM and SCT. We will gather effect sizes and other statistical indicators to assess changes in behavior resulting from different interventions. Additionally, we will consider factors like research methodologies (for example, RCTs and quasi-experiments), the specific groups being studied (such as professional drivers or new drivers), and the tools used for measurement in order to examine both the consistency and variations in outcomes. It is important to evaluate the research quality and potential biases while considering constraints, such as the absence of randomization and reliance on self-reported data collection methods.

## 3 Results

### 3.1 Study selection

**Figure 1:**
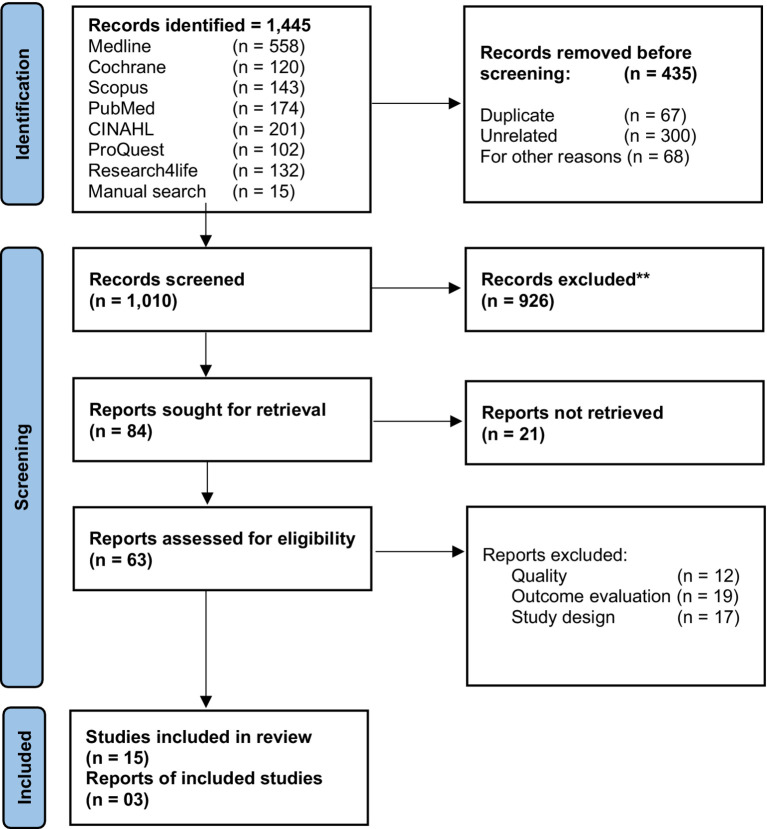
Flow diagram of selection of articles.

The initial search retrieved 1,445 records. After deduplication and screening for title abstracts and full-text, using a predefined list of inclusion/exclusion criteria and assessment for risk of bias, 15 studies that reported information on BCIs and the instruments used to measure the results of these interventions were included in the systematic review (Figure 1).

### 3.2 Study characteristics

The 15 included studies identified eighteen (*n* = 18) types of BCI and nine (*n* = 9) tools for measuring the effectiveness of BCI. The majority of the studies were from Nigeria (*n* = 6), Kenya (*n* = 2), and individual (*n* = 1) studies from Australia, the United Kingdom, Egypt, Iran, Tanzania, Thailand, and Ethiopia. Most studies (*n* = 10) used education and awareness as part of the BCI package. Instruments used to measure the outcome of the BCI vary, but of the eighteen identified, most of the studies (*n* = 7) used questionnaires and (*n* = 7) surveys. The characteristics of BCI are described further in Table 1; the intervention types and summary of outcomes are included in Table 2, and the BCI and Measurement Instruments Used are in Table 3.

**Table 2:** Behavior change intervention and summary of outcomes.

Behavior change intervention	Intervention description	Summary of outcomes
Road safety education and training (32)	Public education through workshops, seminars, and lectures. Driver’s improvement courses. Media campaigns (jingles on radio and television). Distribution of safety pamphlets and posters	Knowledge of traffic codes and road safety signs improved, with knowledge being the strongest predictor of drivers’ attitudes toward the program (*β* = 0.674, *p* < 0.05).
Comparison of fear-based and positively framed road safety films (24)	Virtual reality (VR) vs. 2D film, with participants randomly assigned to one of four conditions (Fear VR, Positive VR, Fear 2D, Positive 2D)	Positive VR and 2D conditions significantly decreased risky driving behaviors. The fear VR condition increased risky driving behaviors, while Fear 2D showed no significant effect. VR delivery produced stronger emotional arousal but was only effective in reducing risky driving when paired with a positive message.
Road safety education and first aid training intervention (25)	Didactic lectures and practical demonstrations on road safety and first aid, facilitated by the Federal Road Safety Commission (FRSC) and Nigeria Red Cross	The intervention group’s road safety knowledge significantly increased immediately after training, but this did not sustain into the fourth month after the intervention. Periodic refresher training is recommended to maintain knowledge retention.
Sticker messaging intervention and radio campaign (27)	Stickers with evocative messages are placed inside minibuses. Messages were designed to encourage passengers to complain to drivers about dangerous driving behaviors. There were different types of stickers (e.g., collective action, individual action). Complementary Intervention: A radio campaign promoting the same message was conducted in certain regions of Kenya.	The sticker intervention reduced insurance claims by 25 to 33%, avoiding approximately 140 accidents and saving about 55 lives annually. Vehicles in the treatment group had average speeds of 1–2 km/h lower than the control group, suggesting reduced reckless driving behavior. The radio campaign did not significantly impact insurance claims or accident rates. Messages promoting collective action were more effective than those promoting individual action in reducing accidents.
Road safety education campaigns and workshops (34)	Group workshops, helmet-wearing campaigns, peer-to-peer interventions, and other educational events.	Helmet-Wearing: Helmet usage increased significantly from 41% (pre-test) to 64% (post-test) in the random group and 63% in the control group. Behavior Change: The most significant changes were in awareness and self-reported helmet-wearing behavior. However, risky behaviors like speeding were less influenced by the intervention, especially among male students with aggressive driving habits.
Education enlightenment campaign (35)	Multimedia-based road safety education, including video slides, films, posters, handbills, and group discussions on traffic rules, safe driving, and emergency handling. The campaign includes collaboration with FRSC and Nigeria Union of Road Workers.	Behavioral Change: The campaign significantly reduced risky driving behaviors like speeding, overloading, and driving under the influence of alcohol. Knowledge Improvement: Drivers showed improved knowledge regarding road safety measures, the dangers of driving while intoxicated, and vehicle maintenance practices.
Traffic safety awareness program (36)	Group discussions, PowerPoint presentations, printed materials, and videos.	Significant improvement in drivers’ knowledge: The post-program test showed a higher knowledge score than the pre-program results, with a statistical significance (*p* < 0.05). Before the program, 57.5% of drivers had poor knowledge, which reduced to 32.5% post-program. Key Findings: Drivers showed improved knowledge about road signs, speed limits, and first aid practices, but misconceptions remained regarding some first aid procedures (e.g., use of coffee powder to stop bleeding).
Classroom theory education and in-car practical training (23)	Combination of theoretical education (classroom) and practical in-car training.	Practical training proved more effective than theoretical education in reducing unsafe driving. Driver attitudes and engagement were vital in changing behaviors, while longer driving hours were linked to more dangerous driving. Human factors had a greater impact on risky behaviors than the program’s specifics, suggesting stricter, more practical approaches for better results.
Information, Education and Communication (IEC) initiative (26)	Face-to-face training sessions and workshops. Educational videos and social media platforms. Publicity materials (e.g., posters, brochures, pamphlets). Mentorship programs by experienced riders.	IEC interventions significantly improved road safety outcomes, with training, educational materials, and media campaigns boosting awareness and promoting behavior change. Regression analysis showed that IEC explained 58.6% of the variance in safety outcomes (R^2^ = 0.586), showing a strong relationship between IEC and improved road safety practices. Motorcyclists preferred face-to-face training and digital platforms, though mentorship programs had mixed results with lower engagement.
Road safety education program (37)	Educational programs covering road traffic codes and safe driving behaviors.	The FRSC education program significantly impacted drivers’ behavior toward road traffic codes and safe driving. However, there was no significant improvement in drivers’ knowledge of road traffic codes and safe driving after the program. A joint effect of drivers’ educational background, the duration of learning, Behavior, and knowledge was observed on their attitudes toward the program.
A qualitative study using a thematic analysis focused on understanding perceptions of risky driving behaviors (33).	In-depth interviews using an open-ended interview guide with 10 public transport drivers, 4 driving school instructors, 3 traffic police officers.	Risky driving behaviors (RDBs) in the region were strongly influenced by gaps in the drivers’ training system, inconsistent law enforcement, the pressure from vehicle owners to maximize profits, and inadequate vehicle maintenance. It was suggested that improving driver education, enforcing consistent road safety laws, and addressing financial pressures on drivers and vehicle owners would help reduce risky driving behaviors.
Health and safety education (31)	In-person talks with visual aids (posters, leaflets) and interactive sessions are held at motor parks.	There was a significant improvement in knowledge, with the mean score rising from 34.4 to 72.3% in the intervention group. Knowledge of highway speed limits increased from 4 to 91.7%. After the intervention, 66.1% of drivers had good overall knowledge, compared to none pre-intervention. However, there was no significant change in self-reported adherence to speed limits, which dropped from 98.4 to 74.9% post-intervention.
SMS text messaging reminders (28)	Three groups, each receiving a different set of messages: (1) social norming messages aimed at emphasizing society’s positive stance on helmet wearing, (2) fear appeal messages that emphasize the dangers of riding without a helmet, and (3) control group messages, which included basic road safety messages unrelated to helmet use.	There was little difference between fear appeal and control group recipients. Subgroup analysis suggests that fear appeal and social norming message types might have been associated with increased helmet use among participants who did not consistently wear helmets at baseline, but this was insignificant (*p* = 0.11 and *p* = 0.07, respectively). Among those who were consistent wearers at baseline, the social norming messages performed better than the fear appeal messages, and this difference reached traditional significance (*p* = 0.03) but was not significant after accounting for multiple tests.
Public education campaign (29)	Public education campaigns through seminars, workshops, and media. Distribution of educational materials such as posters and pamphlets. Use of radio and television jingles	The FRSC public education program positively impacted some aspects of drivers’ behaviors, but significant areas of non-compliance, such as speeding and reckless overtaking, remained prevalent.
3 to 6-week multi-stage driving Behavior change program (P Drivers Program) (30)	Two group sessions and one on-road coaching session, followed by 12 months of maintenance messages. A 3 to 6-week multi-stage driving Behavior change program (P Drivers Program). Surveys were administered at three time points (pre-Program, approximately one-month post-Program and at 12 months after).	The program improved awareness of crash risk factors and intentions to drive safely. However, it did not reduce self-reported crashes or police-reported casualty crashes. Self-reported violations, errors, and risky driving behaviors increased in the intervention group compared to the control group, as did traffic infringements.

**Table 3:** Behavior change intervention and measurement instruments used.

Objective of study	BCI	BCI measurement instrument
To evaluate the commercial vehicle drivers’ perception of the effectiveness of the Federal Road Safety Commission road safety education program on their driving experience, age and behavior toward safety signs on the roads and highways (32).	Public education through workshops, seminars, and lectures. Driver’s improvement courses. Media campaigns (jingles on radio and television). Distribution of safety pamphlets and posters.	A 52-item self-developed questionnaire (Perception and Compliance Road Traffic Rules and Regulation Questionnaire – PCRTRRQ). Focus group discussions
To examine the impact of fear versus positively framed road safety films and traditional technologies (2D) versus emerging technologies (VR) on young drivers’ self-reported risky driving behaviors (24).	Virtual reality (VR) vs. 2D film, with participants randomly assigned to one of four conditions (Fear VR, Positive VR, Fear 2D, Positive 2D).	DBQ: Self-reported risky driving behaviors. Vienna Risk-Taking Test: A standardized behavioral measure of risky driving. Emotional Arousal Scale: Emotional response to the films
To assess the impact of a safety education intervention on knowledge of road traffic accident prevention among drivers in Lagos State, Nigeria (25).	Didactic lectures and practical demonstrations on road safety and first aid, facilitated by the Federal Road Safety Commission (FRSC) and Nigeria Red Cross.	Semi-structured interviewer-administered questionnaire assessing road safety knowledge in three phases: baseline, immediate post-intervention, and 4 months post-intervention
To test the efficacy of evocative messages, delivered on stickers placed inside Kenyan matatus, or minibuses, in reducing road accidents (27).	Stickers with evocative messages are placed inside minibuses. Messages were designed to encourage passengers to complain to drivers about dangerous driving behaviors. There were different types of stickers (e.g., collective action, individual action). Complementary Intervention: A radio campaign promoting the same message was conducted in certain regions of Kenya.	Insurance claims: Used to assess accident rates. GPS data: Recorded speed of vehicles. Passenger surveys: Collected information about passengers’ experiences and exposure to the intervention. Driver behavior observation: Recorded incidents of reckless driving and passenger complaints.
The aim of this research is to keep continuing to manage unsafe driving behavior by road safety education, and to evaluate the Behavior change (34).	Group workshops, helmet-wearing campaigns, peer-to-peer interventions, and other educational events.	Pre-test and post-test questionnaires. Focus groups for qualitative insights.
To examine the effects of Multimedia-based Road Safety Education (MbRSE) on knowledge of and attitude to Safe Driving Behavior (SDB) among inter-state commercial mini-bus drivers in Ibadan metropolis, Nigeria (35).	Multimedia-based road safety education, including video slides, films, posters, handbills, and group discussions on traffic rules, safe driving, and emergency handling. The campaign includes collaboration with FRSC and Nigeria Union of Road Workers.	Evaluation of drivers’ knowledge, attitudes, and behaviors toward road safety practices. No specific instruments were mentioned, but the intervention focused on behavior change and knowledge improvement.
To assess the knowledge regarding traffic safety and first aid measures among the traffic drivers in Alexandria and measure the effect of implementing a traffic safety awareness program on driver’s knowledge regarding traffic safety practices in Alexandria – Egypt (36).	Group discussions, PowerPoint presentations, printed materials, and videos.	Tool I: Demographic characteristics and health status of drivers. Tool II: Driver’s knowledge regarding traffic safety practices assessment tool. Tool III: Driver’s response in traffic accidents and first aid measures checklist
To evaluate the effect of DETP on dangerous driving behaviors of drivers using Structural Equation Modelling (SEM) (23)	Combination of theoretical education (classroom) and practical in-car training.	Self-administered questionnaires focused on Dangerous driving behaviors (e.g., frequency of accidents, driving violations, and near-crashes). Driver’s attitudes toward the education and training programs. Impact of enforcement and education acceptance.
To investigate the influence of information, education and communication on road safety among Boda-boda motorcyclists in Kenyan cities (26).	Face-to-face training sessions and workshops. Educational videos and social media platforms. Publicity materials (e.g., posters, brochures, pamphlets). Mentorship programs by experienced riders.	Semi-structured questionnaires for quantitative data collection. Key informant interviews for qualitative insights
To assess the impact of road safety education by the Federal Road Safety Commission (FRSC) on commercial drivers’ knowledge and Behavior toward road traffic codes and safety driving (37).	Educational programs covering road traffic codes and safe driving Behaviors.	Drivers’ Road Safety Knowledge and Attitudinal Questionnaire (DRSKAQ)
To explore perceptions related to risky driving behavior among public transport vehicle drivers in Debre Markos City, North West Ethiopia (33)	In-depth interviews using an open-ended interview guide with 10 public transport drivers, 4 driving school instructors, 3 traffic police officers.	Thematic analysis was performed using interviews, coded through ATLAS-TI software.
To determine the effectiveness of a post-license road safety education intervention program in terms of increased knowledge and self-reported Behavior among commercial minibus drivers in Lagos, Nigeria (31).	In-person talks with visual aids (posters, leaflets) and interactive sessions, held at motor parks.	Structured, pre-tested, interviewer-administered questionnaires assessing knowledge of road signs, speed limits, and prerequisites for a driver’s license.
To evaluate the comparative impact of two different types of SMS text messaging reminders on motorcycle helmet use (28).	Three groups, each receiving a different set of messages: (1) social norming messages aimed at emphasizing society’s positive stance on helmet wearing, (2) fear appeal messages that emphasized the dangers of riding without a helmet, and (3) control group messages, which included basic road safety messages unrelated to helmet use.	Adherence to helmet use was evaluated by self-report through surveys conducted at baseline, 3 weeks, and 6 weeks.
To assess the effectiveness of the Federal Road Safety Commission public education program in improving drivers’ habit/ behavior on Nigerian roads and highways (29).	Public education campaigns through seminars, workshops, and media. Distribution of educational materials such as posters and pamphlets. Use of radio and television jingles	Drivers’ Perception of the Effectiveness of FRSC Public Education Program Questionnaire (DPEPEPQ): To measure drivers’ views on the program. Drivers’ Observance of Road Traffic Rules and Regulation Checklist (DORTRRC): To record observable driving behaviors
The P Drivers Project was a trial of a behavioral change program developed for and targeted at young Australian drivers in their initial months of solo driving when crash risk is at its highest (30).	Two group sessions and one on-road coaching session, followed by 12 months of maintenance messages.	Self-reported crashes. Police reported casualty crashes. Self-reported attitudes and behaviors (Driver Behavior Questionnaire and other scales). Surveys were administered at three time points (pre-program, approximately one-month post-program and 12 months after).

### 3.3 Results of synthesis

#### 3.3.1 Study designs

Of the 15 studies included in the systematic review, the majority (*n* = 5) used a quasi-experimental study design and (*n* = 4) used RCT. Two other studies used a mixed-method study design, one used an *Ex Post Facto* design, one used a descriptive survey study design, one used structural equation modelling (SEM), and one used a qualitative study design.

#### 3.3.2 Behavior change intervention

The results showed that the majority of BCIs (*n* = 10) used driver’s educational programs and were conducted through various approaches alone or alongside other approaches like workshops or seminars (*n* = 4), printed materials (*n* = 4), media platforms (*n* = 3) and video slides or films (*n* = 3). One study conducted educational BCI alongside practical in-car training. Other studies (*n* = 2) assessed the effectiveness of driver’s educational programs that involve the attitudinal Behavior of drivers toward road signs, driving rules and management of road accident-free environment, which starts and ends in classrooms but did not specifically mention the educational approach. Other BCI that were conducted individually include SMS text messaging (*n* = 1), In-depth interviews (*n* = 1), Road safety films (*n* = 1), sticker messaging (*n* = 1) and Radio campaigns (*n* = 1). The BCIs are summarized in Table 2.

#### 3.3.3 Behavior change theory used

The review result showed a range of behavior change theories used in the studies, either alone or in combination. The major behavior change theories were SCT (*n* = 4), TBP (*n* = 4) and HBM (*n* = 3). TPB focuses on the relationship between beliefs, attitudes, perceived behavioral control, and behavioral intention (21). The SCT emphasizes the influence of observational learning, social reinforcement, and self-efficacy on behavior (22). The HBM proposes that health behavior is influenced by perceived susceptibility, severity, benefits, barriers, and cues to action (21). Other studies (*n* = 4) used a combination of behavior change theories, including a combination of fear appeal theory and SCT (*n* = 1), a combination of social norm theory and collective action theory (*n* = 1), a combination of self-regulation theory and social learning theory (*n* = 1), and a combination of HBM and SCT (*n* = 1).

#### 3.3.4 Outcome measures of behavior change intervention

The outcome measures of the BCI vary in the fifteen studies, with surveys (*n* = 7) and questionnaires (*n* = 8) making up most of the outcome measurement instruments. Other measurement tools include Focus Group Discussions (FGD) (*n* = 1), risk-taking tests, insurance claims (*n* = 1), recorded speed of vehicles (*n* = 1), driver behavior observation (*n* = 1) and self-report crashes (*n* = 1). These outcome measurement tools are used alone or in combination with other tools, particularly the two primary tools, surveys or questionnaires. Surveys used in these reviews include passenger surveys, Knowledge, Attitude and Behavior (KAB) surveys, demographic surveys, self-report surveys and drivers’ perceptions of BCI. Moreover, questionnaires that were used in these reviews include a self-developed questionnaire, a regulation questionnaire, a DBQ, a semi-structured interviewer-administered questionnaire, pre and post-test questionnaires, self-administered questionnaires, Driver’s Road Safety knowledge and Attitudinal Questionnaire (DRSKAQ) and self-reported attitudes and behavior questionnaire. The BCI measurement instruments are summarized in Table 3.

#### 3.3.5 Comparative analysis across studies

This review reveals several patterns in the design, implementation, and effectiveness of BCIs aimed at DDBs across LMICs. While all included studies aimed to address risky behaviors such as speeding, distracted driving, and impaired driving, the methods employed, theoretical foundations, target groups, and contexts varied. This section synthesizes these variations to highlight meaningful comparisons and insights.

##### 3.3.5.1 Intervention type and effectiveness

Educational programs were the most common BCIs used through seminars, campaigns, classroom training, or printed media. These interventions enhanced knowledge and awareness, but the actual behavior change results were not encouraging. Interventions that included practical aspects, such as in-car training (23) or simulation-based training (24) yielded better outcomes in actual driver behavior.

Interventions that included education, media outreach, and community engagement were the most promising (25, 26). These interventions were further reinforced by peer mentorship and social accountability. Single-medium approaches (e.g., standalone posters or brief SMS reminders) were less effective, particularly if they lacked cultural sensitivity or interactivity.

##### 3.3.5.2 Theories used and behavioral outcomes

The review found that the TPB, SCT, and the HBM were the most commonly used behavior change theories. TPB-based interventions successfully changed intention-based behaviors such as wearing a helmet or following speed limits. SCT-based interventions utilized observational learning and social reinforcement (e.g., peer mentors, role modelling) and were effective in areas with strong community networks. HBM-based interventions focused on risk perception and compliance benefits, with results varying based on the content’s engagement level and cultural appropriateness.

The effectiveness of their implementation primarily determined the success of these theories. Interventions that depended solely on superficial constructs (e.g., benefits without action cues) had a reduced impact.

##### 3.3.5.3 Measurement approaches and validity

Self-report surveys and questionnaires were the most commonly used measurement tools. However, social desirability bias and recall inaccuracies limited their effectiveness. Studies utilizing objective measures (e.g., insurance claims, GPS data, traffic records) provided more reliable evidence of behavioral change (23, 27).

Mixed-methods approaches that included surveys, FGDs, or observational tools were used to enhance the findings’ reliability and differentiate between self-reported and actual behavior.

A significant issue identified in all studies is the absence of standardized and validated tools that can be used in LMICs. This highlights a critical research gap in road safety in these countries.

##### 3.3.5.4 Population and contextual constraints

Interventions for commercial drivers and motorcyclists (e.g., minibuses, boda-bodas) were typically tailored to their high-risk occupation. However, the success of these interventions depended on the following contextual factors: enforcement of traffic laws, economic pressures (e.g., incentives to maximize trips), cultural norms surrounding speed, masculinity, and alcohol use, and the availability of ongoing training and infrastructure. For instance, interventions in urban areas with visible law enforcement, such as Egypt and Kenya, had more tangible impacts than those in rural areas with informal transport systems.

In conclusion, this comparative analysis demonstrates that the success of BCIs in LMICs is influenced by the design of the intervention, the cultural environment, the level of theory employed, and the level of measurement used. Interventions that are theory-based, multi-modal, context-sensitive, and well-supported by sound evaluation techniques have a greater potential for enhancing road safety in LMICs.

### 3.4 Risk of bias

The risk of bias assessment revealed that most studies exhibited a low risk of bias across key domains (Selection Bias, Performance and detection bias, Incomplete outcome data attrition bias, Reporting bias, other bias, finance and confounding, and Study design bias). Minor concerns with selection bias, performance, and detection bias were identified in two studies, respectively (24, 28). Minor concerns for incomplete outcomes were also noted in two studies (29, 30). Minor concerns for reporting bias were observed in a few (*n* = 3) studies (28, 30, 31) and study design bias in a few (*n* = 3) other studies (31–33). The overall risk of bias for each study is low. No risks were observed in the majority of the studies (*n* = 8). The Risk of Bias summary is shown in Table 4. These findings indicate that the studies generally had a low risk of bias and thus may not affect the strength of the evidence.

**Table 4:** Risk of bias summary.

Author	Study design	Overall risk of bias level		
		Low risk	High risk	Unclear risk
Abdul- Wahab et al. (32).	Mm-descriptive survey and fgd	X		
Cutello et al. (24).	Randomized control trial	X		
Fowode et al. (25).	Quasi experiment	X		
Habyarimana et al. (27).	Randomized control trial	X		
Jaensirisak et al. (34).	Quasi experiment	X		
Mabayoje et al. (35).	Quasi experiment	X		
Mohamed et al. (36).	Quasi experiment	X		
Nadimi et al. (23).	Structural equation modelling (sem)	X		
Nthoki et al. (26).	Mixed method convergent parallel design	X		
Nwadinigwe et al. (37).	Descriptive survey	X		
Mazengia et al. (33).	Qualitative study using thematic analysis			X
Okafor et al. (31).	Quasi experiment			X
Campbell et al. (28).	Randomized control trial			X
Onuka et al. (29).	Ex-post facto design	X		
Stephan et al. (30).	Randomized control trial			X

The risk levels were categorized according to the criteria established in the respective tools: “low risk” means that there is minimal bias across all domains; “high risk” means that there is one or more domains with serious concerns; and “unclear risk” is used when reporting was insufficient to determine bias.

## 4 Discussion

In synthesizing findings across studies, this review reveals that multiple contextual, theoretical, and methodological factors shape the effectiveness of BCIs. The following section evaluates intervention types, their theoretical foundations, implementation settings, and outcome assessment methods.

### 4.1 Behavior change intervention

The evaluation identified a range of methods to promote safer driving habits by addressing behaviors on the road through various approaches, each with differing levels of impact. Common strategies included public awareness campaigns, hands-on driver training programs, behavior-focused messaging, and peer mentoring initiatives.

Educational initiatives using workshops and media outreach, like radio and television ads, including posters and pamphlets, were prevalent approaches for promoting safe driving (25, 26, 29, 32, 34–37). These programs aimed to increase awareness and enhance understanding of driving practices. Multimedia-based road safety education utilizes films and group discussions in collaboration with road safety authorities. According to research studies, this method has proven effective in engaging a diverse audience (26, 28–30, 36).

The review indicated that most studies found success with a hands-on training method, which combined classroom learning and driving practice to improve skills behind the wheel and follow traffic rules (23, 25, 26, 30, 36). This interactive approach provided guidance and practical experience to help drivers become safer.

Psychological tactics such as using fear appeals and social norms are employed in messaging interventions to influence Behavior. Virtual reality (VR) and traditional 2D films delivered messages highlighting the dangers of risky driving behaviors. At the same time, sticker campaigns in minibuses encouraged passengers to take collective action against dangerous driving (24, 27, 28, 32). Fear-based messaging was also employed to promote helmet use among motorcyclists, highlighting non-compliance risks (26, 28, 34).

Furthermore, the review indicated that peer support initiatives played a role in influencing changes in driver behavior. Seasoned riders and drivers took on the role of mentors to guide and support others directly in influencing their behavior (23, 26, 30, 31, 34). Combining this method with awareness campaigns established a supportive environment to foster lasting behavioral changes (27, 29, 32, 35).

The analysis of included studies indicated that behavioral outcomes were stronger when interventions incorporated educational and interactive elements, such as practical in-car training and peer mentorship. Programs that utilized multiple channels, including theoretical approaches, real-world simulations, and community-based initiatives, produced more reliable results than single-channel methods like posters and SMS reminders. The results suggest that knowledge dissemination needs to be combined with behavior modelling and reinforcement mechanisms, especially when targeting high-risk groups such as commercial drivers and motorcyclists.

### 4.2 Measurement instruments for behavior change interventions

The review highlighted various measurement instruments used to effectively measure BCI’s impact on addressing risky driving habits. Those tools played a role in gauging drivers’ understanding, cognition, and conduct toward diverse BCI campaigns.

The DBQ was frequently used to assess driving behavior effectively by asking individuals to report their driving habits comprehensively. It has proven dependable in research studies and offers a standardized way to evaluate behaviors like speeding tickets and near-miss collisions (23, 25, 26, 30, 31). In addition to the DBQ is the Vienna Risk-Taking Test, which is a widely accepted method for gauging how inclined drivers are toward taking risks on the road (24). The self-reported and observed measures provided insights into how drivers perceive things and their real driving actions.

In addition to self-reports to assess the intervention’s impact on accident rates and driving behavior in real-life situations, researchers utilized objective measures such as insurance claims and GPS data as well (27). These tools enabled tracking vehicle speed and accident claims to gather data that effectively supplements subjective reports. Observed methods like the Driver behavior Observation Checklist captured instances of reckless driving and passenger complaints in real-time, providing direct insights into driving habits (24, 29, 35).

Various semi-structured questionnaires and interview-administered tools were used to evaluate people’s knowledge and opinions on road safety. One of these tools is the Drivers’ Road Safety Knowledge and Attitudinal Questionnaire (DRSKAQ) (23, 35, 37), which successfully measured how well drivers grasped road safety laws and norms throughout the intervention process from the assessment to the follow-up stages. Moreover, the questionnaire known as Drivers’ Perception of the Effectiveness of the FRSC Public Education Program Questionnaire (DPEPEPQ) assessed how drivers perceive the effectiveness of the public education program by the FRSC (29, 32). In contrast to this assessment tool is the Drivers’ Observance of Road Traffic Rules and Regulation Checklist (DORTRRC), which examines drivers’ adherence to road traffic regulations (29).

Additionally, valuable qualitative information was collected by engaging in focus group conversations and conducting interviews with individuals whose responses were organized and examined with the help of tools such as ATLAS TI (25, 31–34). These approaches offered context to the numerical data and shed light on the drivers’ perspectives on behavior change by investigating their attitudes and motivations.

Certain research studies included checklists focusing on health and safety practices, such as the Drivers Response in Traffic Accidents and First Aid Measures Checklist (36), which looked into how drivers were equipped and reacted in accident situations. Compliance with safety protocols, like wearing helmets, was evaluated through self-reports and surveys carried out at the beginning and end of the intervention periods (23–25, 28, 30, 32).

The studies reviewed used a combination of tools, such as surveys (such as DBQ and semi-formal questionnaires), concrete metrics (like GPS information and insurance records), and qualitative methods (like focus groups and interviews). These assessment tools offered a strategy that enabled the consideration of both personal perspectives and factual information in assessing the impact of interventions on improving road safety. The comparative analysis also showed that interventions using both subjective (e.g., surveys) and objective (e.g., GPS data, insurance claims) measures were more effective in assessing actual behavioral change. Programs relying solely on self-report instruments may have underestimated risky behaviors due to social desirability bias or recall bias. In LMIC contexts where access to administrative data may be limited, mixed-method approaches seem to provide a more balanced and credible evaluation of intervention outcomes.

### 4.3 Application of behavior change theories in road safety interventions

Various behavior change theories were used in the BCIs to address unsafe driving behaviors effectively. Many key behavior change theory frameworks guided the development and execution of these interventions by focusing on psychological and social factors that influence behavior change.

One of the most frequently applied theories was the TPB. The theory played a role in numerous initiatives, such as the ones carried out by the Federal Road Safety Commission (FRSC) in Nigeria and various public awareness campaigns aimed at drivers (28, 29, 31, 34). TPB focuses on three core components: attitudes, subjective norms, and perceived behavioral control, all of which influence an individual’s intentions to engage in safer driving practices (38). When interventions based on the TPB were implemented to alter drivers’ perceptions and motivations related to driving habits by addressing their beliefs about the outcomes of their actions and reinforcing social norms concerning road safety, they successfully promoted changes in behavior and compliance with traffic regulations (28, 29, 31, 34). This approach proved effective in initiatives targeting the enhancement of safe driving practices among drivers, as seen in studies like the Impact of Road Safety Education in Delta State and University Drivers in Ibadan research projects.

The HBM was frequently used in interventions aimed at raising drivers’ understanding of the risks of accidents and the advantages of practising safer driving habits (38). This model was involved in projects like the Federal Road Safety Commission Training in Jigawa State and the Traffic Safety Awareness Program in Alexandria. It also played a key role in media campaigns targeting Boda Boda motorcyclists in Kenya (32, 33, 36, 37). These initiatives sought to enhance drivers’ awareness of risks by highlighting the benefits of adhering to traffic rules for their safety and that of their communities. By addressing challenges and highlighting the advantages of applying the HBM (a model that helps predict health-related behaviors), these campaigns effectively motivated drivers to adopt habits such as wearing helmets and following speed limits (33, 36, 37).

The SCT has also been instrumental in developing programs that center on peer learning and mentorship initiatives, underscoring the value of observation learning, social influence and reinforcement mechanisms (38). BCIs, like the Media Campaign for Boda Boda Motorcyclists and the Adult Education Campaign for Mini Bus Drivers, used SCT by providing chances for drivers to witness and copy behaviors exhibited by their peers or seasoned riders (23–26, 33, 35). This approach promoted behavior change and fostered a supportive social environment in which safe driving practices could be reinforced. Similarly, Social Learning Theory (SLT), closely related to SCT, was employed in interventions like the P Drivers Program in Australia and the Driving Education Programs in Delta State (30, 37). The interventions based on SLT emphasized demonstrating role models and offering feedback to drivers so they could learn and modify their behaviors by observing and receiving reinforcement. These measures impacted novice drivers’ behavior, outlook, and road safety (30).

Some interventions use the Fear Appeal Theory to elicit reactions and prompt individual behavioral shifts. For instance, in research contrasting Virtual Reality with 2D Film presentations, fear-inducing messages emphasize the perils of unsafe driving habits, intending to encourage participants to avoid such behaviors (24). Nonetheless, the impact of fear appeal differed based on the framing of the messages and the setting in which they were presented (39, 40). Additionally, social norm theory and collective action theory were employed in Kenya’s Large-Scale BCI, which leveraged social norms and collective responsibility to influence passenger and driver behaviors (27). These theories emphasized the power of societal expectations and group action in promoting compliance with road safety rules.

Finally, the Self-Regulation Theory and aspects of the Transtheoretical Model (TTM) were applied to help individuals set goals and adjust their behaviors through self-observation and feedback mechanisms. This approach, though less commonly cited, has proven impactful in driving incremental improvement (30). They provided valuable frameworks for guiding behavior change through self-monitoring and gradual progression toward safer driving habits (38).

The effectiveness of TPB, SCT, and HBM appears to depend on the implementation depth. Interventions that extend beyond surface-level messaging and target behavioral intentions, self-efficacy, or perceived risk are more likely to influence real-world behavior. This suggests that merely referencing a theory is insufficient; instead, integrating theory into the design and delivery mechanisms of interventions is essential for effectiveness.

### 4.4 Predominant study designs and their relation to other vital variables

This review showed that different study designs were employed to assess BCIs to enhance road safety. RCTs, Quasi-Experimental Designs, Mixed Methods Approaches, and Qualitative Studies were the most common. The type of intervention and target population, alongside the intervention’s theoretical framework, underpinned each design choice, all of which played distinct roles in enhancing our understanding of behavior change outcomes.

RCTs were commonly employed to assess the effectiveness of interventions by comparing outcomes between randomized intervention and control groups. Cutello et al. in the UK (24), Habyarimana et al. in Kenya (27), Campbell et al. in Tanzania (28), and Stephan et al. in Australia (30) applied RCTs to test interventions rigorously. These research studies focused on populations ranging from everyday drivers to motorcycle taxi drivers and new drivers. Theories like Fear Appeal Theory, SCT, TPB and Self-Regulation Theory guided the interventions (24, 27, 28, 30), enabling researchers to evaluate outcomes such as risky driving behaviors, emotional responses, adherence to helmet use, and traffic violations.

RCTs have shown success in offering strong evidence of the effectiveness of interventions by evaluating theory-based strategies in controlled environments (41). Quasi-experimental designs, on the other hand, were commonly applied in situations where randomization was not feasible for community or educational initiatives (42). This approach was notably implemented in research conducted by authors like Fowode et al., Jaensirisak et al., Mabayoje et al., Mohamed et al., and Okafor et al. (25, 31, 34–36). These interventions mainly focused on university drivers, motorcyclists and commercial minibus drivers. The SCT, the HBM, and the TPB all focused on drivers’ understanding of road safety regulations and their adherence to them to reduce driving behaviors in society. For instance, research conducted by Mohamed et al. And Okafor et al. Examined individuals’ awareness of traffic safety regulations and willingness to follow road rules. These studies demonstrated how quasi-experiments could be effectively used to evaluate changes in behavior in real-life scenarios (31, 36).

Furthermore, Mixed methods designs were chosen in research where both quantitative and qualitative perspectives were necessary for comprehending behaviors and attitudes (43). For instance, Thoki et al. used a convergent design to assess the road safety habits of motorcyclists in Kenya (26). Mixed methods proved valuable for interventions based on SCT, emphasizing learning and reinforcement as critical elements in driving behavioral changes (43). These designs integrated data like road safety knowledge scores with drivers’ subjective viewpoints to offer a holistic understanding of behavioral shifts.

Mazengia et al. delved deeply into people’s thoughts and feelings about driving using qualitative approaches (33). These studies employed a combination of HBM and SCT to explore drivers’ motivations and beliefs about road safety. The thematic analysis brought out an understanding of the psychological aspects that influence risky driving behaviors, providing additional insights that complemented the numerical results (44).

The contextual challenges faced by LMICS, such as limited enforcement, economic pressures on drivers, and road infrastructure issues, emerged as critical factors influencing intervention success. Interventions located in urban areas with more robust regulatory frameworks tended to yield more quantifiable results. This underscores the importance of context-sensitive design and the integration of structural supports, such as enforcement partnerships or ongoing training programs, to enhance the impact of interventions.

### 4.5 Implications for policy and practice

The review indicates that BCIs in LMICs are most effective when they are theory-based, culturally adapted, and reinforced over time. Policymakers should invest in multi-component interventions, prioritize community engagement, and utilise validated outcome measurement tools. Additionally, integrating behavioral interventions into national road safety strategies could reduce traffic-related injuries and fatalities in resource-constrained settings.

### 4.6 Limitations

Some studies had flaws in the methods, such as lack of randomization, small sample sizes and uncertain bias levels in specific domains. Therefore, these limitations may affect the reliability and generalizability of the findings. Moreover, comparing the studies was made difficult by the level of variance in the types of intervention, the measurement tools and the outcomes evaluated. There was a lack of standardized measures for assessing changes in Behavior across different studies, which might affect result consistency. Finally, the evaluation could be affected by a bias in publication since research that yields negative results is less likely to be published.

## 5 Future research

For future studies, researchers should consider exploring a more comprehensive range of driver populations with diverse cultural and economic backgrounds to enhance the applicability and inclusiveness of results. Moreover, combining qualitative data through Mixed methods design provides valuable perspectives. It would be beneficial for research to adopt this method to capture both tangible impacts and environmental factors that impact changes in Behavior. Moreover, future studies should aim to develop and adopt standardized tools for measuring Behavior change outcomes to facilitate comparison across studies and improve data consistency.

## 6 Conclusion

This systematic review sought to identify the most common BCIs (BCIs) targeting DDBs and to examine the instruments used to measure these interventions’ outcomes. The review showed that public education campaigns, practical driver improvement programs, behavioral messaging interventions, and peer-to-peer mentorship were the most prevalent interventions applied across diverse driving populations. These BCI were usually based on established behavior change theories, such as the TPB, the HBM and the SCT. Different types of tools were used to measure these BCI, including self-reported instruments like the DBQ, guided surveys as well as objective data sources such as GPS tracking and insurance claims records to assess road safety aspects of BCIs effectiveness in various studies together with qualitative methods like focus groups and thematic analyses to gain a holistic perspective, on the subject.

## Data Availability

The original contributions presented in the study are included in the article/supplementary material, further inquiries can be directed to the corresponding author.
